# Infraumbilical Abscess Due to Meckel’s Diverticulum Co-occurring With a Urachal Remnant: A Case Report

**DOI:** 10.7759/cureus.110426

**Published:** 2026-06-07

**Authors:** Ayano Tsukizaki, Hirofumi Tomita, Akihiro Shimotakahara, Kentaro Matsuoka

**Affiliations:** 1 Department of Surgery, Tokyo Metropolitan Children’s Medical Center, Tokyo, JPN; 2 Department of Pathology, Tokyo Metropolitan Children’s Medical Center, Tokyo, JPN

**Keywords:** infraumbilical abscess, meckel diverticulum, meckel’s diverticulum, omphalomesenteric duct remnant, preperitoneal abscess, urachal cyst, urachal remnant

## Abstract

Meckel’s diverticulum is the most common congenital anomaly of the gastrointestinal tract, and urachal remnants, another congenital anomaly, arise from the incomplete involution of the fetal urachus. Although both conditions have been studied extensively, their co-occurrence is rare and may complicate the diagnosis, particularly if an infection is present. A 22-month-old male patient presenting with abdominal pain was found to have an infraumbilical abscess in the preperitoneal space. Initial imaging studies demonstrated a urachal abscess extending toward the bladder. Following antibiotic therapy, elective surgery was performed collaboratively by pediatric urologists and pediatric surgeons to manage a concurrent inguinal hernia. Intraoperatively, Meckel’s diverticulum with a mesenteric band connected to the umbilicus was identified in addition to a urachal cyst, prompting an en bloc resection of the abscess, urachal cyst, and Meckel’s diverticulum. Histopathological analysis demonstrated that the abscess most likely originated in the omphalomesenteric remnant rather than the urachal remnant. Infraumbilical abscesses can originate in omphalomesenteric duct remnants as well as urachal remnants. Clinicians should consider the possibility of the co-occurrence of multiple congenital anomalies in a patient with an infraumbilical abscess to ensure complete surgical treatment.

## Introduction

Meckel’s diverticulum, the most common congenital anomaly of the gastrointestinal tract, has an incidence of 0.6%-4% [1]. Incomplete obliteration of the omphalomesenteric duct may result in a patent omphalomesenteric duct, umbilical sinus, umbilical polyp, mesodiverticular band, or Meckel’s diverticulum. Although most patients remain asymptomatic throughout their life, these anomalies can cause life-threatening complications, such as bleeding, bowel obstruction (including intussusception, volvulus, and internal hernia due to a mesodiverticular band), inflammation, or perforation [1,2].

The urachus remnant, a tubular structure that extends from the dome of the fetal bladder to the umbilicus, is another congenital anomaly associated with the umbilicus. Prenatally, it normally undergoes involution until it becomes the median umbilical ligament [3]. Incomplete regression results in urachal anomalies, which are classified as a patent urachus, urachal cyst, urachal sinus, or vesicourachal diverticulum [3,4]. Urachal remnants occur in approximately 1% of pediatric patients and are often found incidentally [3]. Although many cases remain asymptomatic, infection is the most common complication and typically manifests as lower abdominal pain, fever, umbilical discharge, or a preperitoneal abscess adjacent to the bladder [3]. Ultrasound is the first-line imaging modality for evaluating these symptoms in children. These congenital remnants have similar symptoms, making an accurate diagnosis difficult. Furthermore, the co-occurrence of urachal remnants and anomalies of the omphalomesenteric duct has rarely been reported to date [5-9], further hampering accurate diagnosis when inflammatory changes obscure the anatomical origin.

## Case presentation

**Figure 1:**
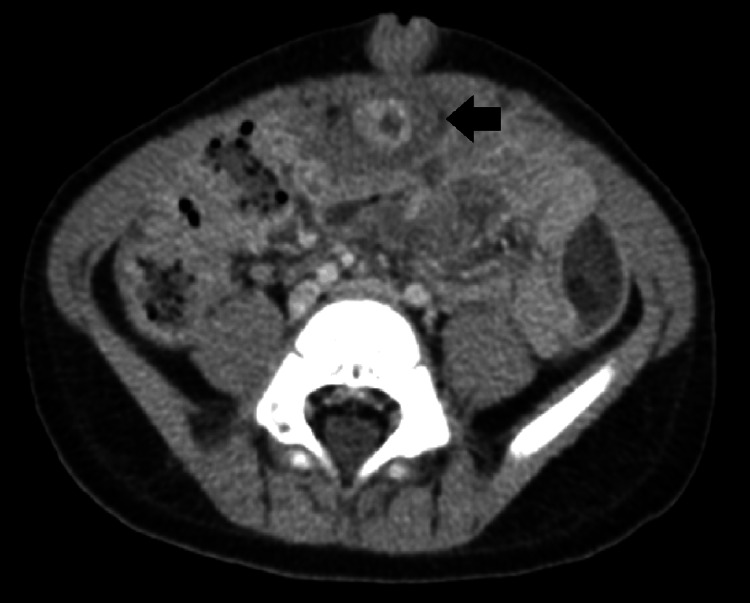
Computed tomography (CT) CT demonstrated an infraumbilical abscess (black arrow).

A 22-month-old male patient presented to a different hospital with complaints of abdominal pain, irritability, and gait disturbance. He was admitted for management, but the inflammatory response intensified over several days. The physicians there performed contrast-enhanced computed tomography (CT) and suspected panperitonitis due to a perforated Meckel's diverticulum (Figure 1), and the patient was subsequently transferred to our hospital.

Physical examination at our hospital revealed tenderness in the infraumbilical region. Ultrasonography demonstrated a preperitoneal abscess measuring approximately 22 × 20 × 28 mm and extending toward the bladder. Inflammation of the mass hampered the identification of intestinal structures connected to the umbilicus and the abscess; therefore, Meckel’s diverticulum was not suspected preoperatively. The patient initially received intravenous antibiotics under a presumptive diagnosis of urachal abscess and his pain resolved promptly. Follow-up ultrasonography showed a residual abscess and elective surgery was planned following the resolution of the inflammation. Two weeks before surgery, left inguinal hernia developed. Consequently, a combined surgical approach was planned to repair both anomalies simultaneously by pediatric urologists and pediatric surgeons three months after the initial episode.

At the beginning of the procedure, pediatric urologists performed a cystoscopy, which found no evidence of a diverticulum or fistula within the bladder. Pediatric surgeons then performed laparoscopic, percutaneous, extraperitoneal closure for the left-sided inguinal hernia, then excised the urachal remnants with the assistance of the urological surgeon. A circular incision was made at the umbilicus to core out the remnant while maintaining the integrity of the urachus. The laparoscopic findings revealed the infraumbilical abscess, which appeared as a mass firmly adhering to the bilateral umbilical arteries through the formation of dense, inflammatory adhesions.

Adjacent to this mass, another cord-like structure, approximately 1 cm in diameter, was identified extending from the umbilicus. Upon exteriorization, this structure was found to be connected to the small intestine. This finding led to the definitive diagnosis of Meckel’s diverticulum. Interestingly, the diverticulum itself appeared to be without acute inflammation at the time of surgery. The urachal cyst was located close to the dome of the bladder. Saline infused through a urethral catheter confirmed the absence of communication between the bladder and the cyst, a feature consistent with a urachal cyst rather than a fistula. The cyst and the bilateral umbilical arteries were then excised.

**Figure 2:**
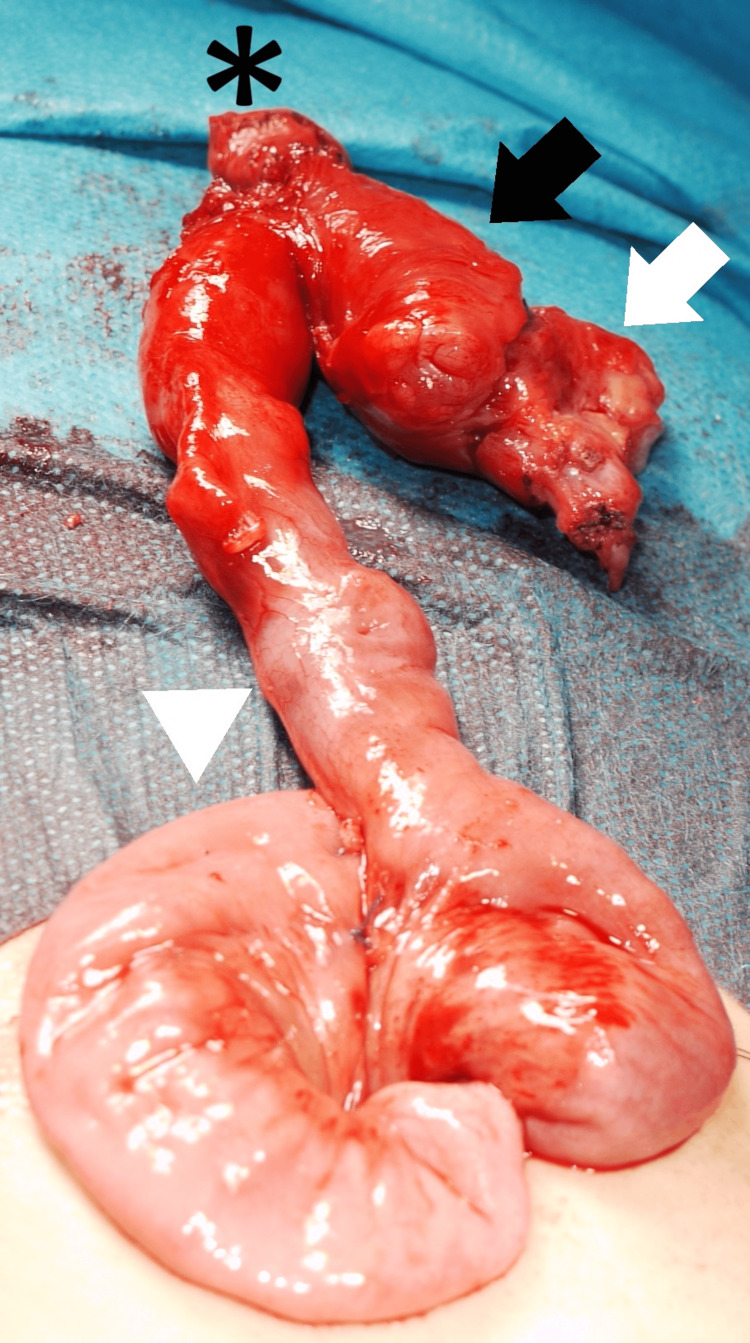
Surgical view of the two remnants The intraoperative images demonstrate the co-occurrence of the omphalomesenteric remnant and the urachal remnant, both connected to the umbilicus. The black arrow indicates the infraumbilical abscess, the white arrow indicates the urachus and bilateral umbilical arteries, the asterisk indicates the umbilicus, and the arrowhead indicates the Meckel's diverticulum.

The Meckel’s diverticulum had a mesenteric band connected to the umbilicus. This band was resected, and a wedge resection of the diverticulum was performed, followed by repair using Albert-Lembert sutures. The abscess, the urachal cyst, and the Meckel’s diverticulum were resected en bloc (Figure 2).

The patient’s postoperative course was uneventful, and he was discharged on postoperative day nine. 

**Figure 3:**
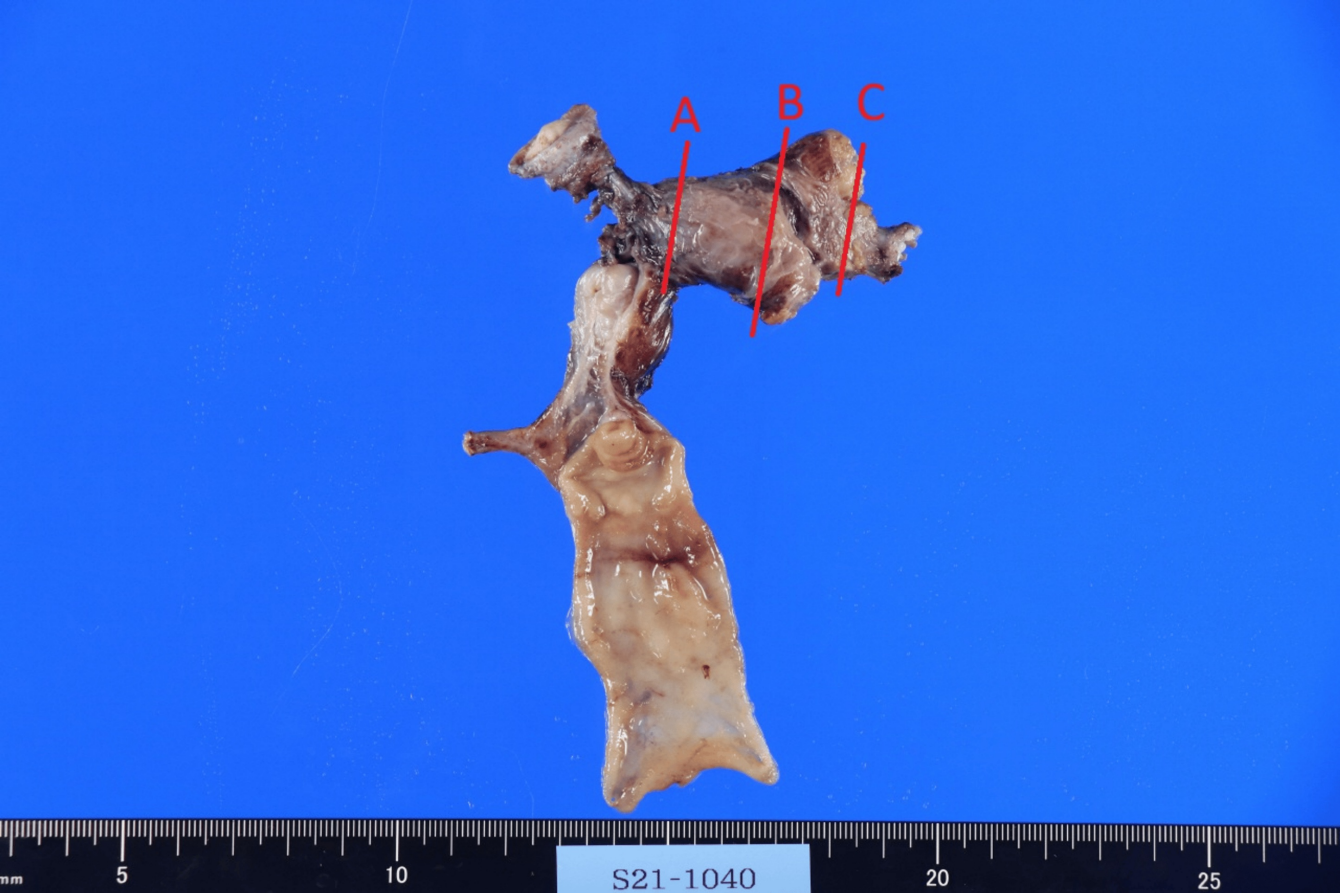
Gross appearance of the resected specimen Pathological examination found the two congenital remnants. The infraumbilical abscess (B) was adjoined to the omphalomesenteric remnant (A) and the urachal cyst (C).

Pathological examination confirmed that the preperitoneal abscess was connected to both the urachal cyst and the omphalomesenteric remnant (Figure 3). 

**Figure 4:**
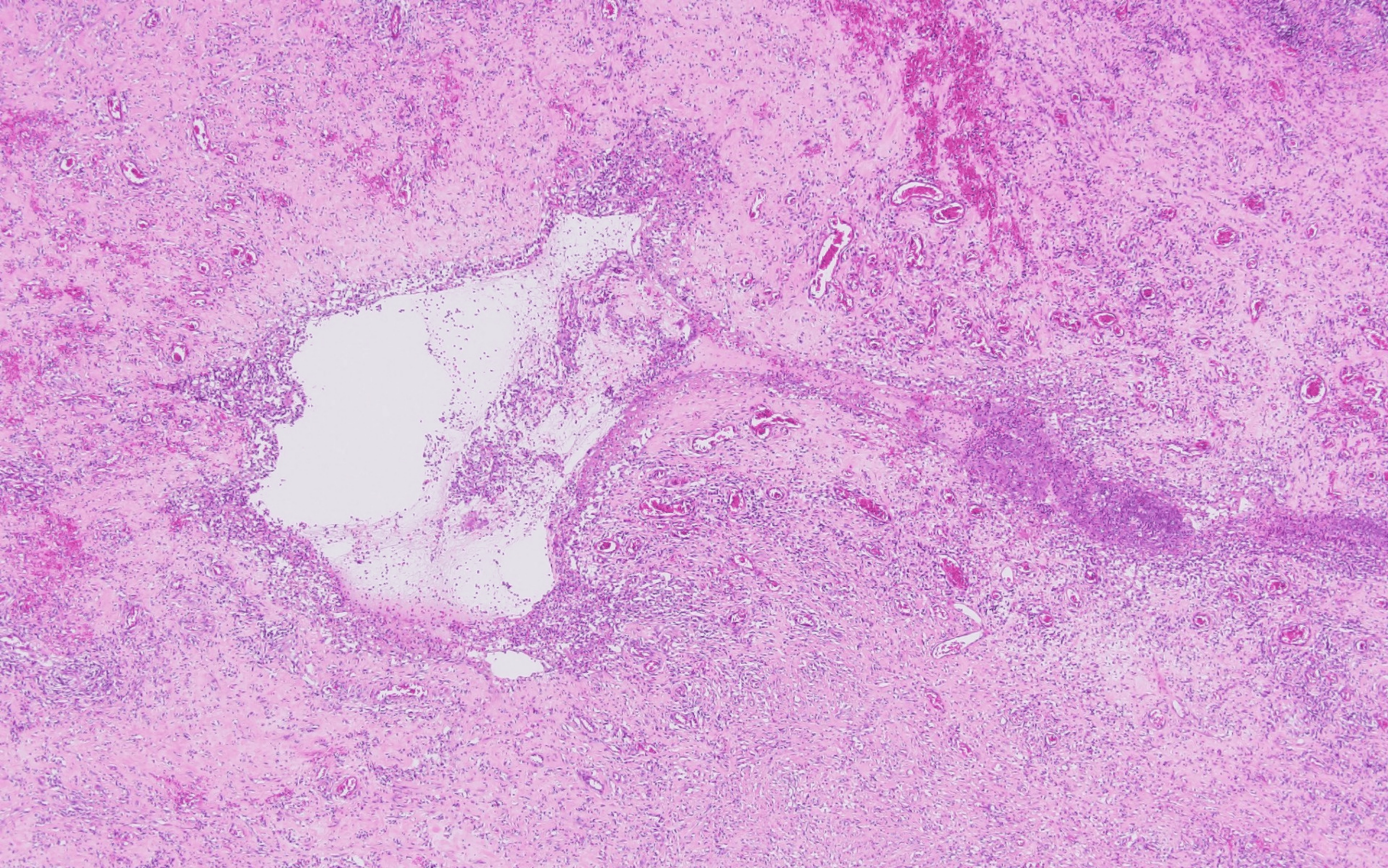
Histopathological findings of the umbilical abscess The abscess lacked an organized epithelium, and the primary structure had dissipated through inflammation in the sections stained by hematoxylin and eosin (H&E). This photograph shows a cross-section along the line B present in Figure 3.

Although the primary structure of the abscess had dissipated (Figure 4), histological clues to the origin of the inflammation remained.

**Figure 5:**
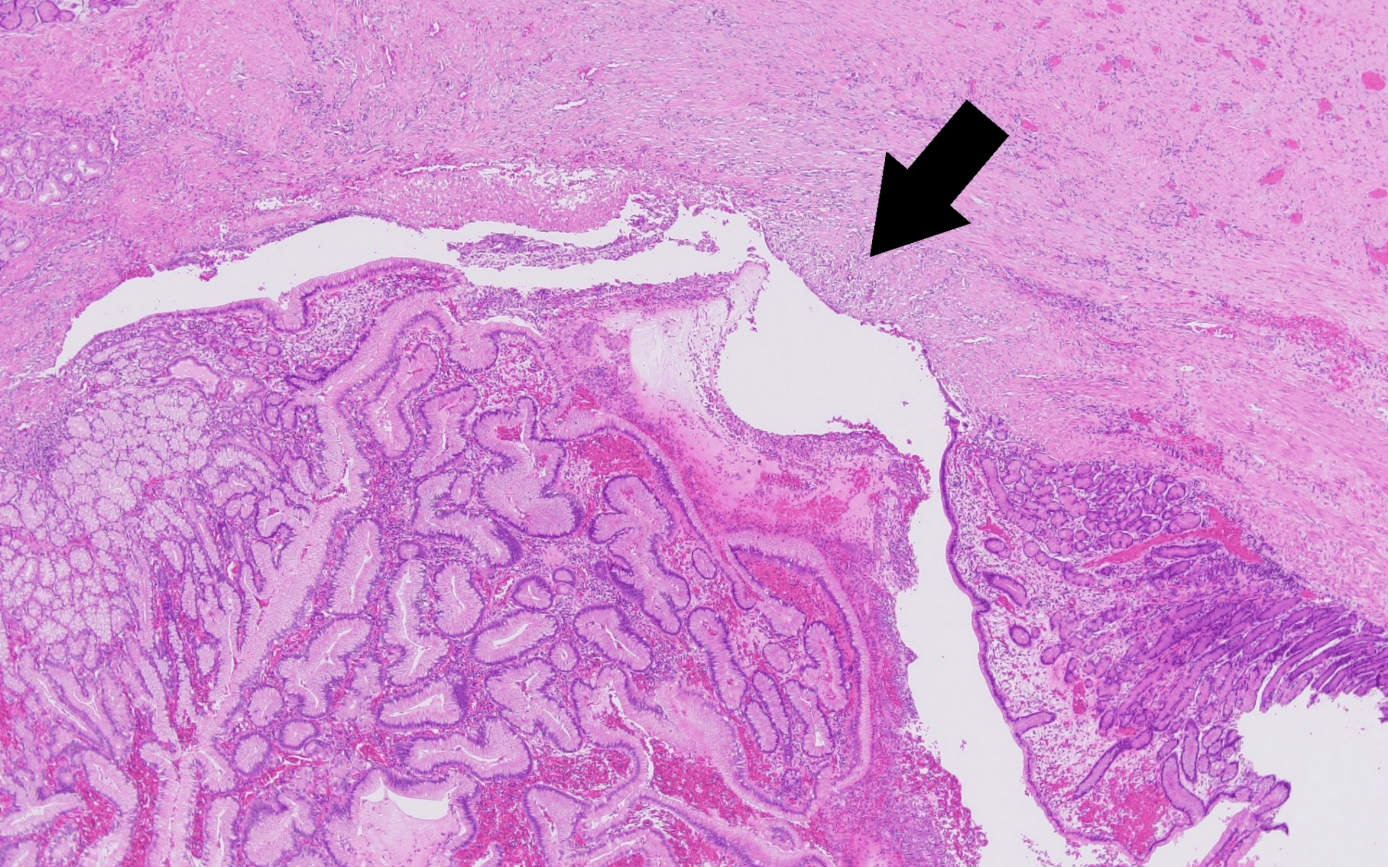
Photomicrograph of the omphalomesenteric remnant H&E staining demonstrated a partial ulcer (black arrow) within the omphalomesenteric remnant due to ectopic gastric mucosa. This image corresponds to the section shown in Figure 3A.

The omphalomesenteric remnant was lined by ectopic gastric mucosa with fundic-type glands, showing ulceration and epithelial exfoliation (Figure 5), consistent with inflammation originating from the luminal side.

**Figure 6:**
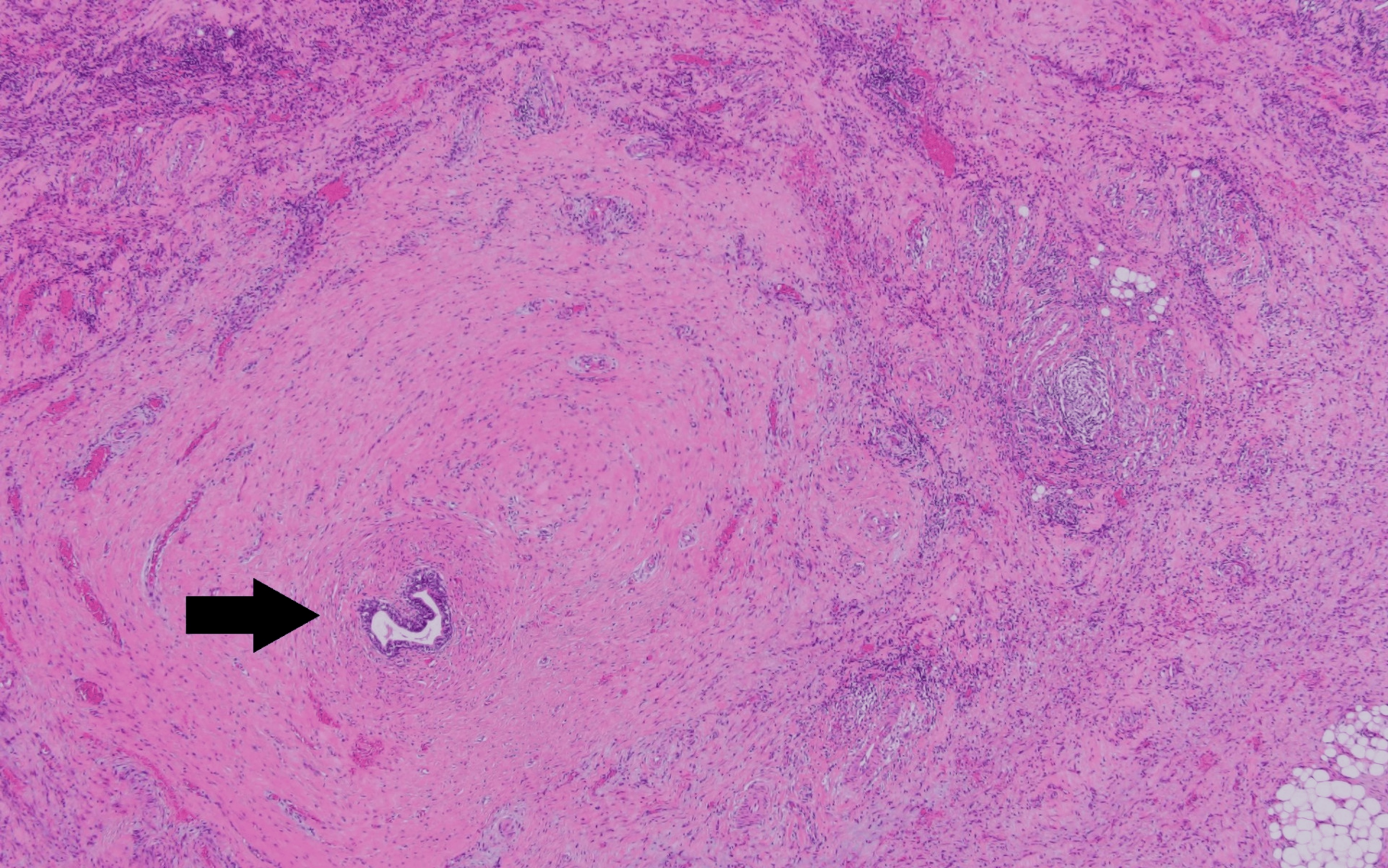
Histopathological findings of the urachal remnant The urachal remnant (black arrow) was surrounded by inflammatory cell infiltration primarily on its outer surface (H&E staining). This image shows the section in Figure 3C.

In contrast, the urachal remnant, lined by flattened to cuboidal epithelium, exhibited inflammatory cell infiltration predominantly on the external surface without evidence of mucosal injury (Figure 6). 

These findings support the interpretation that the infraumbilical abscess was more likely caused by the omphalomesenteric remnant rather than the urachal remnant. The Meckel’s diverticulum itself did not show acute inflammation, and the omphalomesenteric duct was partially duplicated. No external fistula to the skin surface was observed in either remnant.

## Discussion

Meckel’s diverticulum is the most common malformation of omphalomesenteric duct remnants, with bleeding from ectopic gastric mucosa being its most common symptom. Technetium-99m pertechnetate scintigraphy is widely used to diagnose this condition because of its affinity for ectopic gastric tissue. Kusumoto et al. reported that the preoperative diagnostic accuracy of Meckel’s diverticulum was 88% in patients presenting with bleeding whereas it was only 11% in those with other symptoms [10]. Therefore, diagnosing Meckel’s diverticulum in the absence of bleeding remains challenging.

In our case, the patient presented with the atypical symptom of a preperitoneal abscess caudal to the umbilicus which was associated with a patent urachal remnant extending toward the bladder. Stefanopol et al. reported a case of preperitoneal abscess originating in a Meckel’s diverticulum that was misdiagnosed as an infected urachal cyst [11]. Ali et al. also described a perforated Meckel’s diverticulum anterior to the urinary bladder [12].

Unlike Meckel remnants, urachal remnants can become infected and form an abscess between the umbilicus and the bladder. Given this possibility, it was challenging to identify the Meckel’s diverticulum rather than the urachal remnant in this case as the primary origin.

In addition to this unusual feature, the co-occurrence of these two types of remnants is exceedingly rare and complicates the diagnosis. Penninga et al. reported an infant in whom both remnants were diagnosed preoperatively via ultrasonography [5]. In some instances, only the omphalomesenteric duct remnant was diagnosed preoperatively, with the urachal remnant being discovered later [6,7]. Other cases required emergency laparotomy, during which both remnants were identified incidentally [8,9].

In the present case, the surgery was successful despite the intraoperative discovery of Meckel’s diverticulum because a pediatric urologist and a pediatric surgeon performed the surgery together. This multidisciplinary collaboration was highly beneficial, as a preperitoneal abscess may originate in either a urachal or an omphalomesenteric remnant, and due consideration must be given to the possibility of their rare co-occurrence to ensure complete, surgical treatment.

## Conclusions

This case demonstrates that an infraumbilical preperitoneal abscess, while typically associated with urachal remnants, can also originate from omphalomesenteric duct remnants. Both types of remnant can co-occur, albeit rarely, and thus complicate the clinical presentation and obscure the primary source of infection. Careful radiological assessment and a high index of suspicion are essential for accurate diagnosis, particularly in the absence of typical symptoms, such as gastrointestinal bleeding. In complex cases like the present one, a multidisciplinary, surgical approach facilitating definitive diagnosis and complete resection may be instrumental in producing a favorable outcome.
